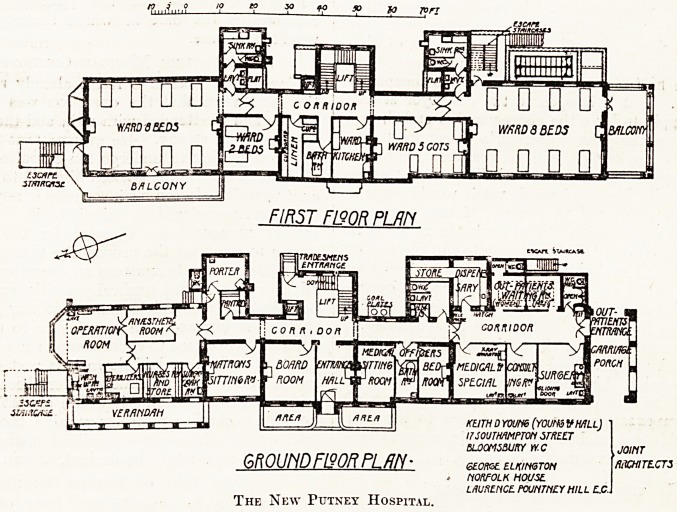# An Excellent Type for the Small General Hospital

**Published:** 1913-03-29

**Authors:** 


### HOSPITAL ARCHITECTURE AND CONSTRUCTION.

### An Excellent Type for the Small General Hospital.

The new Putney Hospital was founded by the late Mr. Henry Chester, who a few years since left the sum of about £75,000 for the endowment of a general hospital for the parish of Putney. The sum of some £20,000 was subscribed by the residents of the district for economical and efficient manner, while at the same time the design embodies all the features requisite to a modern hospital from the medical and hygienic points of view.

The joint architects were Mr. Keith D. Young, F.R.I.B.A. (of Messrs. Young and Hall), and Mr

The New Putney Hospital.

the erection and equipment of the new hospital, and the site on Putney Common was the gift of Sir William John Lancaster, J.P. As will be seen from the accompanying plans, the hospital is a building of three storeys. On the ground floor at the south end and facing Lower Richmond Road is a small but complete out-patients' department, with access from a covered carriage porch. In the centre is the main entrance and hall, adjoining which is the board-room, the resident medical officers' quarters, and the matron's sitting-room, while at the north end is a very complete operation suite.

The whole of the first floor, which is approached by an easy stair with a central bed lift, forms the hospital proper; the accommodation comprising two eight-bed wards, each opening on to a large balcony, a separation ward for two beds, a children's ward for five cots, a central ward kitchen, bathroom, linen store, and the sanitary annexes.

On the second floor are the kitchen, scullery, and offices, and the apartments of the staff, the central service lift serving all floors.

The whole building is a most excellent type for a modern small general hospital, the centralisation of the service and the allocation and arrangement of the various departments allowing of the administration and service being performed in the most

George Elkington, F.R.I.B.A.; and the contractors Messrs. Johnson, of Wandsworth.

## Figures and Tables

**Figure f1:**